# Identification of Sensitive Serum microRNA Biomarkers for Radiation Biodosimetry

**DOI:** 10.1371/journal.pone.0057603

**Published:** 2013-02-25

**Authors:** Naduparambil Korah Jacob, James V. Cooley, Tamara N. Yee, Jidhin Jacob, Hansjuerg Alder, Priyankara Wickramasinghe, Kirsteen H. Maclean, Arnab Chakravarti

**Affiliations:** 1 Department of Radiation Oncology, Comprehensive Cancer Center, The Ohio State University, Columbus, Ohio, United States of America; 2 Department of Molecular Virology, Immunology, and Medical Genetics, The Ohio State University, Columbus, Ohio, United States of America; 3 Center for Systems and Computational Biology, The Wistar Institute, Philadelphia, Pennsylvania, United States of America; 4 NanoString Technologies, Seattle, Washington, United States of America; NIH, United States of America

## Abstract

Exposure to ionizing radiation through environmental, occupational or a nuclear reactor accident such as the recent Fukushima Daiichi incident often results in major consequences to human health. The injury caused by radiation can manifest as acute radiation syndromes within weeks in organs with proliferating cells such as hematopoietic and gastrointestinal systems. Cancers, fibrosis and degenerative diseases are also reported in organs with differentiated cells, months or years later. Studies conducted on atom bomb survivors, nuclear reactor workers and animal models have shown a direct correlation of these effects with the absorbed dose. Physical dosimeters and the available radio-responsive biologics in body fluids, whose responses are rather indirect, have limitations to accurately evaluate the extent of post exposure damage. We have used an amplification-free, hybridization based quantitative assay utilizing the nCounter multiplex platform developed by nanoString Technologies to compare the levels of over 600 miRNAs in serum from mice irradiated at a range of 1 to 12 Gy at 24 and 48 hr time points. Development of a novel normalization strategy using multiple spike-in oligonucleotides allowed accurate measurement of radiation dose and time dependent changes in serum miRNAs. The response of several evolutionarily conserved miRNAs abundant in serum, were found to be robust and sensitive in the dose range relevant for medical triage and in patients who receive total body radiation as preparative regimen for bone marrow transplantation. Notably, miRNA-150, abundant in lymphocytes, exhibited a dose and time dependent decrease in serum, which we propose as a sensitive marker indicative of lymphocyte depletion and bone marrow damage. Our study has identified several markers useful for evaluation of an individual’s response by minimally invasive methods, relevant to triage in case of a radiation accident and evaluation of toxicity and response during and after therapeutic radiation.

## Introduction

Management of radiological causalities that could occur from natural calamities, failures in operational safety mechanisms of nuclear power plants or even a terrorist attack require immediate intervention from emergency responders and medical personnel. The damage caused by a meltdown can be catastrophic as it could release large amounts of radioactivity that quickly affects the environment and the health of surrounding population. Recent events involving the Fukushima Daiichi nuclear reactor have shown the unfortunate and immediate dangers posed by accidental radiation exposure. Nuclear exposure management protocols include rapid dose assessment for the affected population and identification of the individuals who require immediate medical attention. Development of robust biomarkers based on an individual’s biological response is crucial for accurate assessment of the level of exposure and making important medical decisions. A personalized assessment will allow evaluation of an individual’s physiological response to radiation damage. The calculated LD50 for humans exposed to total body irradiation is in the range of 4.0 to 4.5 Gy and the dose range at which supportive care will be effective is narrow. Therefore, development of biomarkers for fast and accurate dose assessment is critical. Moreover, an individual’s response varies depending on many confounding factors such as immune status, age and genetics. These factors will ultimately determine a person’s apparent response to exposure, and in some cases victims may not immediately exhibit visible signs of radiation damage. Therefore, physical dosimetry alone or the available protein markers such as cytokines have limitations to accurately estimate the dose and response of an individual.

Acute effects (Acute Radiation Syndromes, ARS) will manifest themselves as Hematopoietic, Gastrointestinal (GI) and Cerebrovascular syndromes. Studies have shown that individuals exposed to an intermediate dose (5–8 Gy) could die within a few weeks due to GI syndrome. Lower doses (2–5 Gy) that are not immediately lethal but compromise the hematopoietic system can increase susceptibility to infection and death within months if supportive care is not provided on time [1], [2], [3], [4], [5], [6]. In addition, several of the victims who show little or no signs of acute radiation sickness could find themselves dealing with late effects in the form of cancer, pulmonary fibrosis and chronic or progressive heart and kidney diseases. Epidemiological studies on survivors of the Hiroshima and Nagasaki A-bombs and Chernobyl nuclear accident showed an increased incidence of various cancers and cardiovascular diseases [6], [7], [8]. Thus, development of biomarkers capable of accurately estimating the dose absorbed is important for identifying the individuals at risk for acute as well as late effects. Understanding the dose exposed will help in the making of medical decisions and timely administration of immune-modulators and mitigators. Development of such biomarkers will also help understand the response and toxicity in patients receiving therapeutic radiation, particularly for those who receive total body irradiation as a preparative step for bone marrow transplantation.

Over the last several years, there have been attempts to estimate the radiation dose exposed using hematological, biochemical and cytogenetic parameters [9], [10], [11]. Several protein markers such as C-reactive protein, amylase, and cytokines such as transforming growth factors have been investigated for their potential as biodosimeters [11]. These protein markers, however, have large inter-individual variations; the readouts are indirect and fluctuate as a result of common variables such as inflammation and infection [9], [11]. Currently, lymphocyte depletion kinetics, clinical observation, and the dicentric chromosome (DC) assay are used for post exposure dose assessment. Lymphocyte depletion analysis requires repeated measurements over a prolonged period of time and the DC assay is highly technically involved and labor intensive [9], [12]. Therefore, there are needs for identification of novel markers that are sensitive to incremental changes in dose, and are robust and stable for days after exposure and repeatedly assayable in a non-invasive or minimally invasive manner.

microRNAs (miRNAs) are non-coding RNAs of 19–22 nucleotides that were originally identified as regulators of gene expression by inducing cleavage of their target mRNA or blocking translation through base pairing to partially complementary sequences [13]. miRNAs regulate diverse cellular processes including development, proliferation and differentiation, as well as various disease progressions [14]. In addition to their roles in post-transcriptional gene regulation, miRNAs in body fluids are proposed and have been assessed as biomarkers for various physiological responses and pathological stages [15], [16], [17], [18], [19], [20]. Earlier studies have detected miRNAs in a range of body fluids such as serum, plasma and urine, and miRNAs are relatively stable due to their smaller size and being protected in exosomes [21], [22]. However, the current PCR based methods used for evaluation of miRNA in body fluids have limitations. Because several miRNAs are present in low quantities, PCR based detection and quantification often requires pre-amplification of the template and a higher number of amplification cycles, which compromises the reliability of the measurements [23]. To circumvent this problem, we have used a digital amplification-free quantification and comparison method [24] which enabled us to evaluate the relative abundance of individual miRNAs in the serum samples and develop a panel of sensitive biomarkers for radiation biodosimetry.

## Materials and Methods

### Animal Studies

For animal studies involving acute single dose exposure, we used 8–9 week old *Mus musculus.* Male inbred mice (Strains CBA/J and C57BL/6, Jackson Laboratories) were co-housed (five per standard cage) and fed *ad libitum*. Mice were exposed to total body gamma radiation (TBI) using GammaCell@40 irradiator (Cesium −137 Source) at a dose rate of 1.1 Gy/min). For each radiation dose (0, 1, 2, 4, 6 and 8 Gy) and time point (24 and 48 hrs) a minimum of five animals were used. Control animals were sham-exposed. For investigating the effect of fractionated dose, 15 animals were exposed to X-rays (in 2 Gy fractions) from a RS-2000 Biological Irradiator at a dose rate of 1 Gy/min. All the animal experiments were done with strict adherence to the institutional guidelines established and approved by the Ohio State University Animal Care and Use Committee (Permit number: 2011A00000029).

Blood was collected by submandibular bleeding or by cardiac puncture. Following coagulation (1 hr at room temperature), serum was separated using microtainer tubes (BD Biosciences) by centrifugation at 10,000 g for 10 min, and then frozen at −80°C. RNA was extracted using the Qiagen miRNA easy kit following the manufacturer’s protocol. miRNAs were isolated from serum samples collected from 4–5 animals for each time points, and samples with high levels of hemolysis were excluded from analysis. In a typical isolation procedure, 100 µl serum was used. After lysis using QIAzol reagent, 4–20 pg synthetic oligonucleotides (spike-in oligos) Osa-miRNA-414, Cel-miRNA-248, At-miRNA-159a (Integrated DNA Technologies) were added prior to extraction. RNA was eluted in 100 µl water and concentrated to 20 µl and 3 µl was used for each assay for profiling using nanoString Technologies’ multiplexed nCounter platform. The platform incorporates fluorescent barcodes together with a digital readout for single-molecule imaging [24]. It does not involve reverse transcription; instead the technology relies on sequence-specific probes to digitally measure miRNA abundance. This hybridization based amplification-free method allows processing of multiple samples, comparing and quantifying the number of molecules even of low abundance. The spike-in oligos allow a volume and quantity based normalization for detection of even small changes in individual miRNAs.

### miRNA Expression Profiling

The digital multiplexed nanoString nCounter mouse miRNA expression assay (nanoString Technologies) was performed with 10–30 ng total RNA isolated from a net volume of 20 µl serum as input material. Small RNA samples were prepared by ligating a specific DNA tag (miR-tag) onto the 3′ end of each mature miRNA according to the manufacturer’s instruction. These tags serve several purposes: they normalize the wide range of melting temperatures (Tms) of the miRNAs, provide a template to facilitate the use of the nanoString dual probe system, enable single base pair discrimination and specificity of highly homologous miRNA family members, and identify each miRNA species. Excess tags were removed by restriction digestion at 37°C. Hybridizations were carried out by combining 5 µl of each miRNA-miRTag sample with 20 µl of nCounter Reporter probes in hybridization buffer and 5 µl of nCounter Capture probes (for a total reaction volume of 30 µl) overnight at 65°C for 16–20 hrs. Excess probes were removed using two-step magnetic bead based purification on the nCounter Prep Station (nanoString Technologies). Abundances of specific target molecules were quantified on the nCounter Digital Analyzer by counting the individual fluorescent barcodes and assessing the target molecules. For each assay, a high-density scan encompassing 600 fields of view was performed. The data was collected using the nCounter Digital Analyzer after taking images of the immobilized fluorescent reporters in the sample cartridge with a CCD camera.

### Data Analysis

miRNA data analysis was performed using the nSolver software analysis, freely available from nanoString Technologies. The serum miRNA profiling data was normalized using the average signals obtained from three spike-in oligos, and miRNAs that gave significant hybridization signals were used for downstream analysis. ANOVA was performed with a cutoff p-value of 0.05 to identify a set of miRNAs that had the highest difference in means across samples. Coefficient of variance across samples was also performed with a cutoff of 0.4 and overlapping sets of miRNAs from the above two methods were selected as the most significant set. R software was used for the analysis.

## Results

### Optimization of Methods for Quantitative Analysis of Serum miRNAs

**Figure 1:**
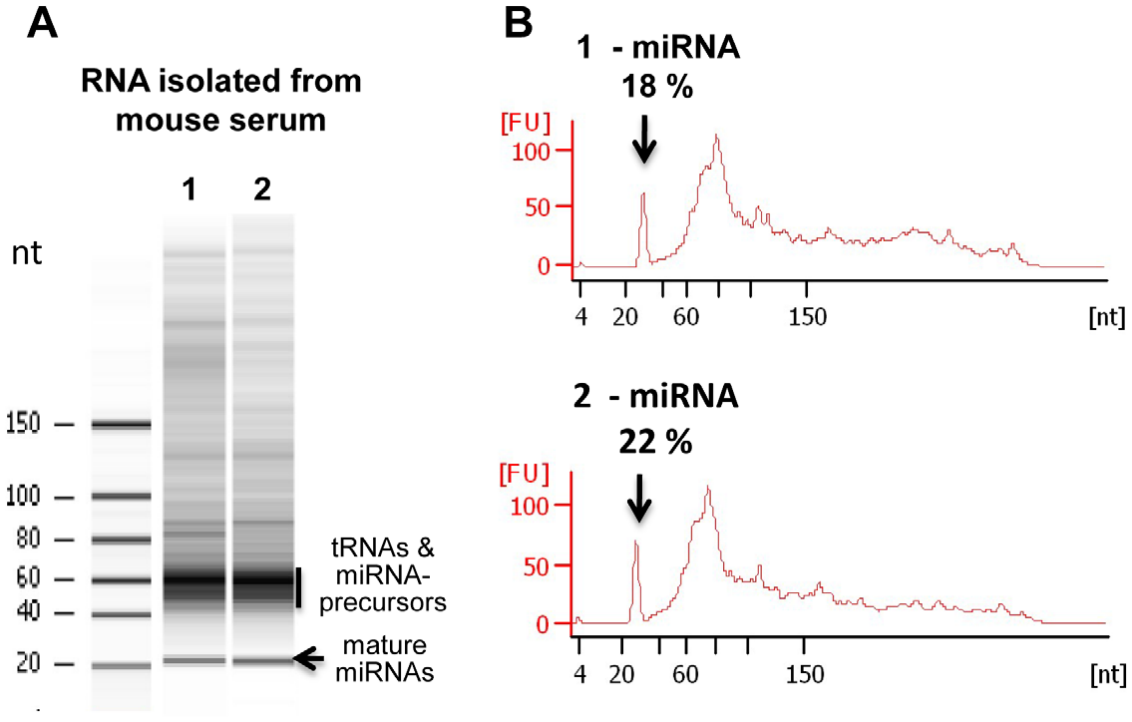
Validation of the integrity of RNA isolated from serum using an Agilent small RNA Bioanalyzer. A: Gel images show the integrity of RNA isolated from two serum samples. B: Densitometry traces were used to quantify and compare the relative abundance of various small RNAs.

The digital multiplexed nanoString nCounter mouse miRNA expression assay was performed on total RNA isolated from 20 µl of serum usually containing a total amount of 10–30 ng of RNA. The nCounter multiplex platform is capable of detecting approximately 600 mouse specific miRNAs, four housekeeping genes and three non-mammalian miRNAs: Osa-miRNA-414, Cel-miRNA-248 and At-miRNA-159a. During RNA isolation, synthetic oligonucleotides (spike-in oligos) corresponding to Osa-miRNA-414, Cel-miRNA-248 and At-miRNA-159a were included as controls allowing the normalization of samples. The amounts of spike-in oligos were optimized by comparing their counts with that of endogenous miRNAs in serum samples. The optimal amount of spike-in oligos for normalization was identified to be 0.5–2 pg in each reaction (data not shown). The inclusion of probes hybridizing to the house keeping genes enabled us to further identify and separate preparations with cellular RNA contaminations. The optimized method allowed detection of changes in serum miRNAs that are specific to changes in physiological and treatment conditions, such as response to radiation. The purity and integrity of the RNA recovered from serum samples was validated on an small RNA bioanalyzer (Figure 1A). miRNAs were found to represent 18–22% of total serum RNA preparations (Figure 1B).

**Figure 2:**
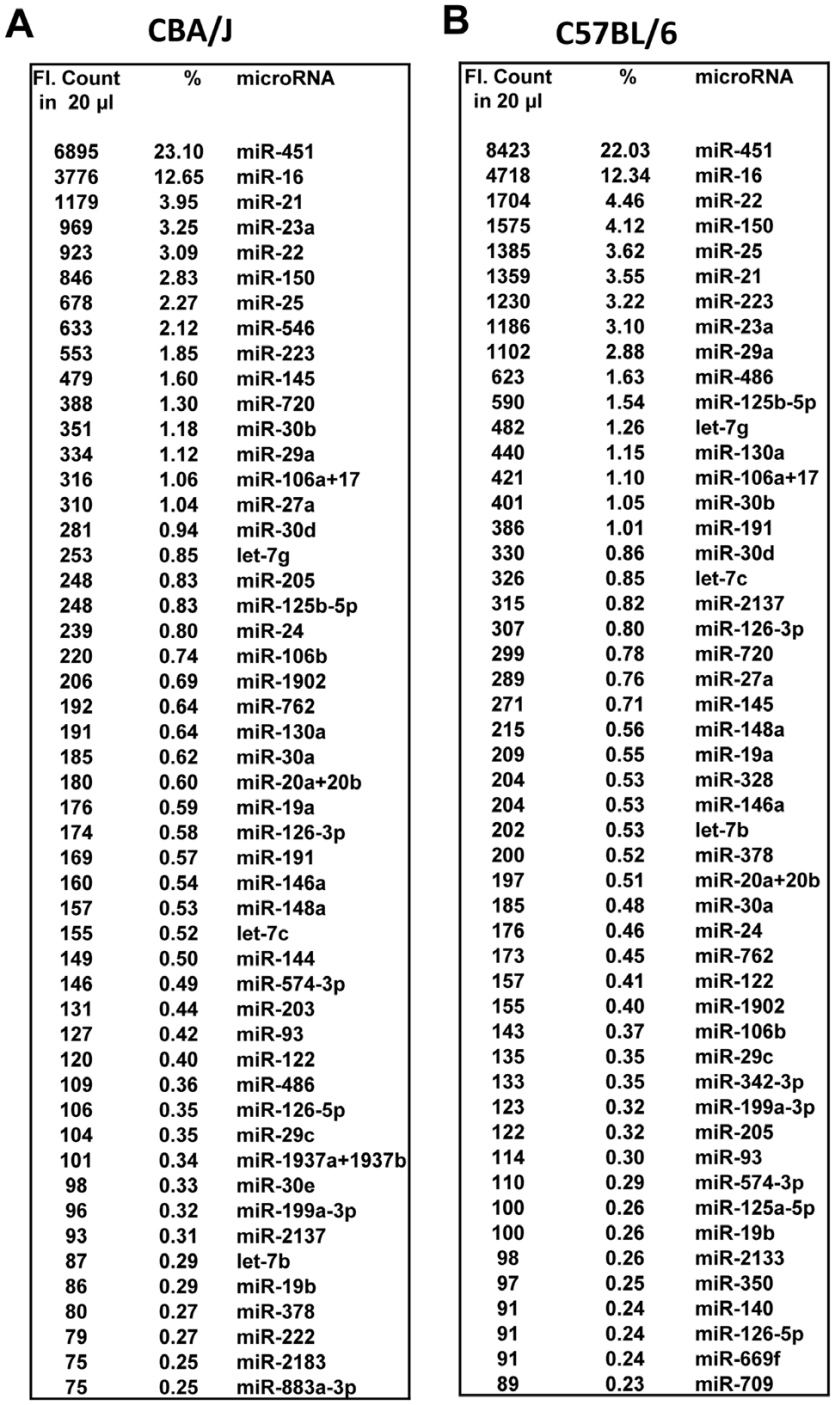
A list of miRNAs detected in mouse serum, arranged in their order of abundance. The average signals (counts) detected from a total of 20 µl serum RNA preparations from three different control mice belonging to two different mice strains (A: CBA/J and B: C57BL/6) are presented. The miRNAs are listed in the order of abundance.

**Figure 3:**
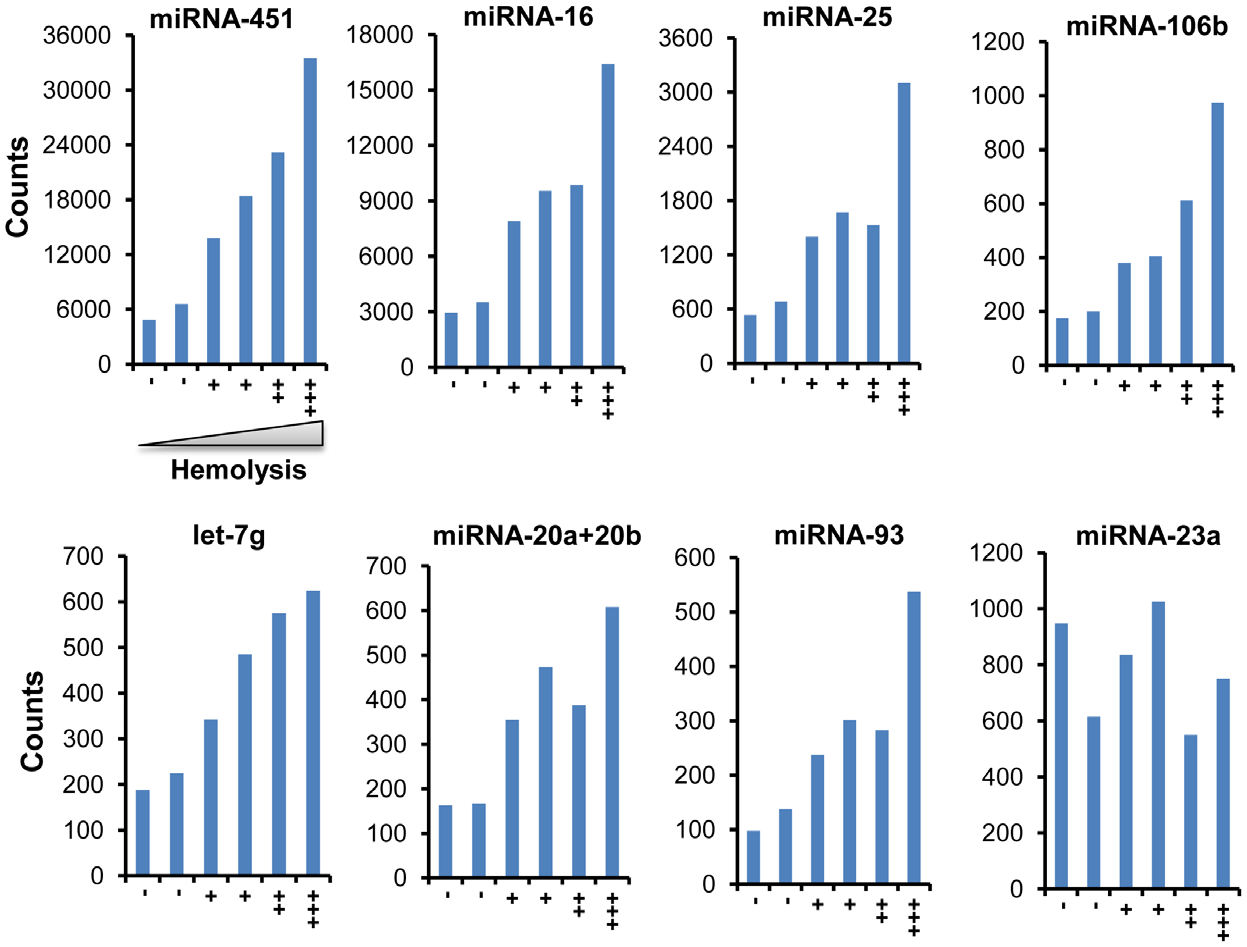
Identification of miRNAs derived from red blood cells that potentially contaminate serum samples due to increased levels of hemolysis. The X- axis shows samples with increasing levels (+) of hemolysis and the Y-axis shows the actual counts of several miRNAs that are altered with an increase in hemolysis.

The nCounter expression profiling conducted on total RNA isolated from mice serum samples identified 88 miRNAs with high confidence. miRNA-451 was found to be the most abundant in serum preparations, contributing to 22–23% of total miRNAs (Figure 2). miRNA-16 ranked second, representing ∼13%. Analysis of serum samples from a minimum of three animals from each of the two strains of mice (CBA/J and C57BL/6) showed similar results. Several evolutionarily conserved and functionally significant miRNAs, such as miRNA-150, miRNA-21, miRNA-29a and miRNA-23a, were also detected in serum samples [25], [26], [27], [28], [29]. Given the abundance of miRNA-451 and miRNA-16 in serum, we investigated the feasibility of using these as endogenous normalizers by comparing their signals with that of spike-in oligos. However, because of the abundance of these miRNAs in red blood cells, even a small level of hemolysis was found to skew the results. Therefore, these endogenous markers were not used as biological normalizers. Furthermore, comparison of samples with increasing levels of hemolysis enabled us to identify additional markers that potentially originating from the lysis of red blood cells. These include miRNA-25, miRNA-106b, let-7g and miRNA-93 (Figure 3), while the level of miRNA-23a was not increased in samples with higher levels of hemolysis. Thus, parallel analysis of samples normalized with multiple controls allowed us to identify markers that are specific and sensitive to radiation treatment.

### Radiation Dose Dependant Changes in Serum miRNA Profile Following Single Acute Dose

Using the nCounter multiplex assay, miRNAs in serum samples from control and irradiated animals collected 24 hrs after 1, 2, 4, 6 and 8 Gy total body irradiation (TBI) were compared. In order to minimize experimental error, irradiation, serum collection, RNA isolation, miRNA profiling, and normalization were done in parallel with controls and treatment groups. Samples with traces of cellular RNA contamination (with counts of 30 or above for any of the four housekeeping genes) were excluded from the analysis. Samples with high levels of hemolysis observed visually or based on relative abundance of miRNA-451 (>23%), miRNA-16 (>13%) correlating with increase in miRNA-25, miRNA-106b, let-7 g and miRNA-93 were also excluded from analysis.

**Figure 4:**
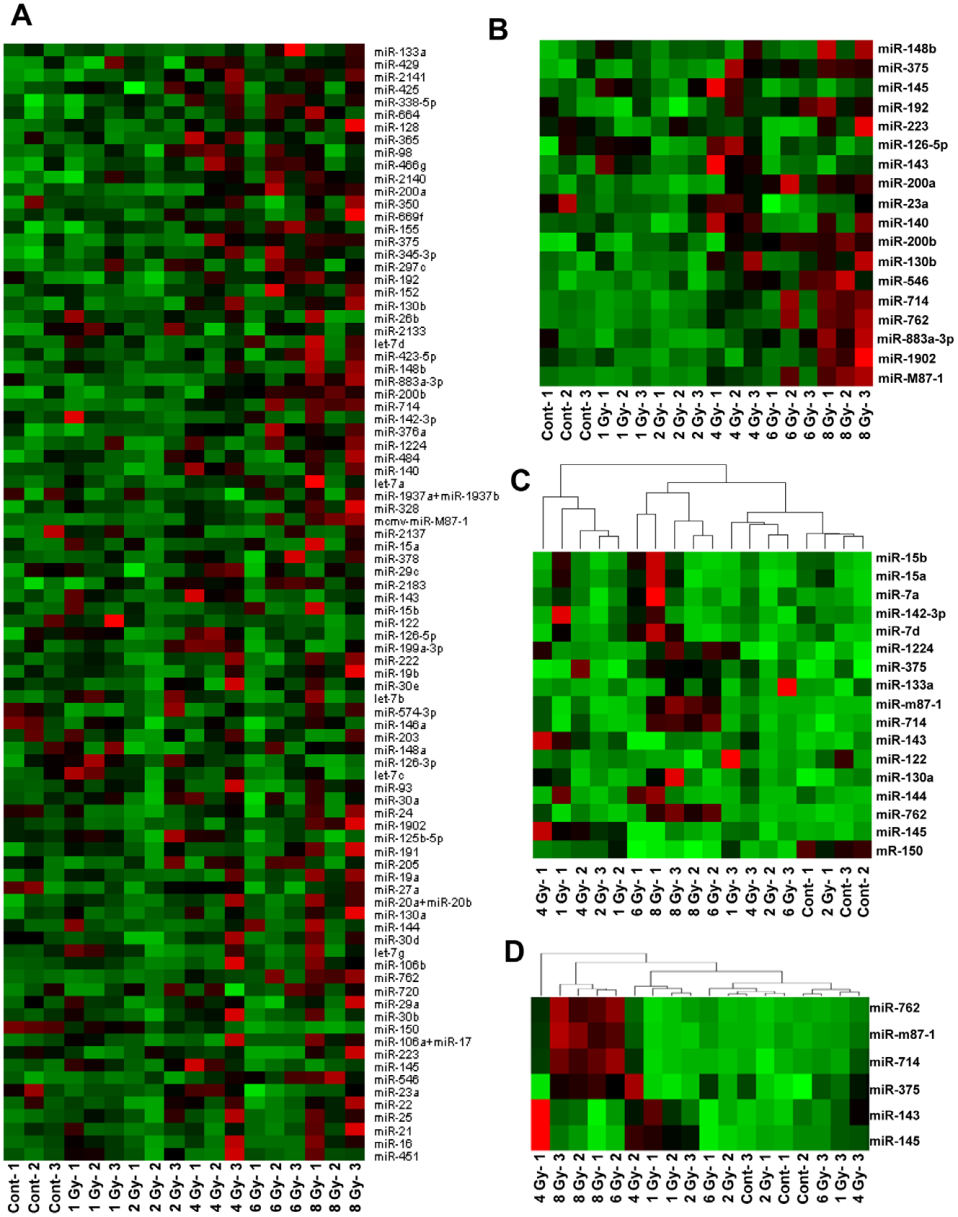
Identification of differentially expressed serum miRNAs with varying doses of radiation at 24 hrs. A: Heat map generated from the actual counts for 88 miRNAs detected in serum. B: Heat map showing variations in a panel of 18 radio-responsive miRNAs, identified by ANOVA with a cutoff p-value of 0.05. C: Dendrogram with a panel of markers identified with coefficient of variance across samples with a cutoff value of 0.4. D: Overlapping set of miRNAs from ANOVA and CV. Red marks high and green marks low expression.

The relative changes of 88 miRNAs detected in serum samples were evaluated for their radiation dose dependent changes (Figure 4A). Changes were observed in several miRNAs distinguishable from irradiated versus controls and between different doses of radiation (Figure 4B). At first, ANOVA was performed with a cutoff *p*-value of 0.05 to identify a set of miRNAs that had the highest difference in means across samples. Next, the coefficient of variance was calculated with a cutoff of 0.4 (Figure 4C). Finally, an overlapping set of miRNAs from the above two methods was selected as the most significant and responsive set (Figure 4D). Several markers were found clustering with specific dose or dose range indicating a clear radiation biodosimetry potential.

**Figure 5:**
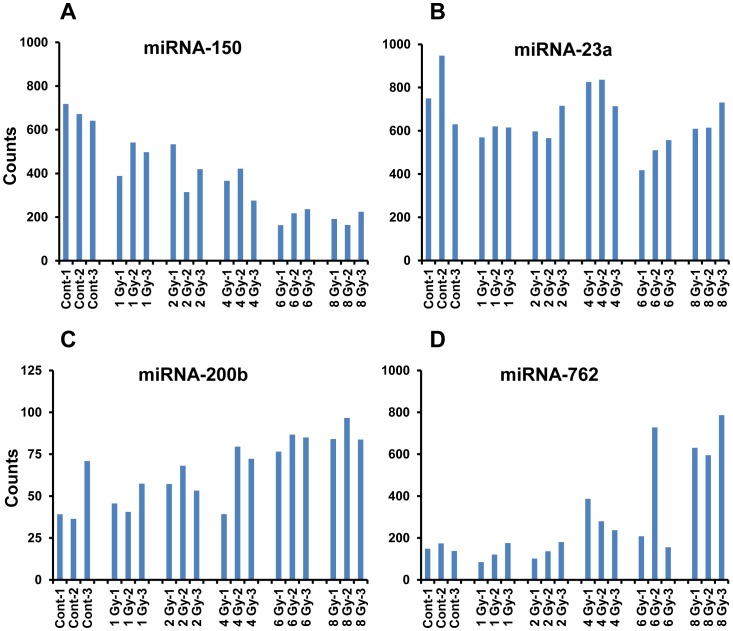
Analysis of fold variations of selected serum miRNA biomarkers with a clear dose response. Histograms show variations in the fluorescent counts detected in the nCounter multiplex assay, plotted against treatment. The counts obtained after normalization using multiple spike-in oligos were plotted for individual animals. A: The dose dependent depletion of serum miRNA-150 at 24 hrs (p-values: 1 Gy- 0.0164, 2 Gy- 0.0191, 4 Gy- 0.0026, 6 Gy- 0.0001, 8 Gy- 0.0001). B: Counts from a non-responsive molecule miRNA-23a, comparable to that of miRNA-150 in control animals. C and D: Radiation induced increase in miRNA-200b and miRNA-762 (p-values, miRNA-200b:1 Gy- 0.7172, 2 Gy-0.4193, 4 Gy- 0.4231, 6 Gy- 0.0421, 8 Gy- 0.0296; miRNA-762∶1 Gy- 0.4061, 2 Gy- 0.1675, 4 Gy- 0.0324, 6 Gy- 0.3139, 8 Gy- 0.001).

Selected radiosensitive miRNAs identified from cluster analysis were further investigated for their dose and time dependent changes. In order to evaluate the robustness of the response of each individual marker, we chose to plot the normalized fluorescence counts from individual animals that received varying doses of radiation (Figure 5). miRNA-150 was identified as a robust radio-responsive serum biomarker, with a clear dose response in all animals compared 24 hrs after radiation (Figure 5A). A decrease in levels of miRNA-150 was evident even in animals that received 1 Gy radiation, which further decreased with increasing dose (2, 4, 6 and 8 Gy). Molecules that exhibited an increase in their serum levels after radiation exposure include miRNA-200b and miRNA-762, and these changes were more pronounced in animals that received higher doses (Figure 5C, 5D). miRNA-23a, whose counts in controls are comparable to that of miRNA-150 was used as another control (Figure 5B).

**Figure 6:**
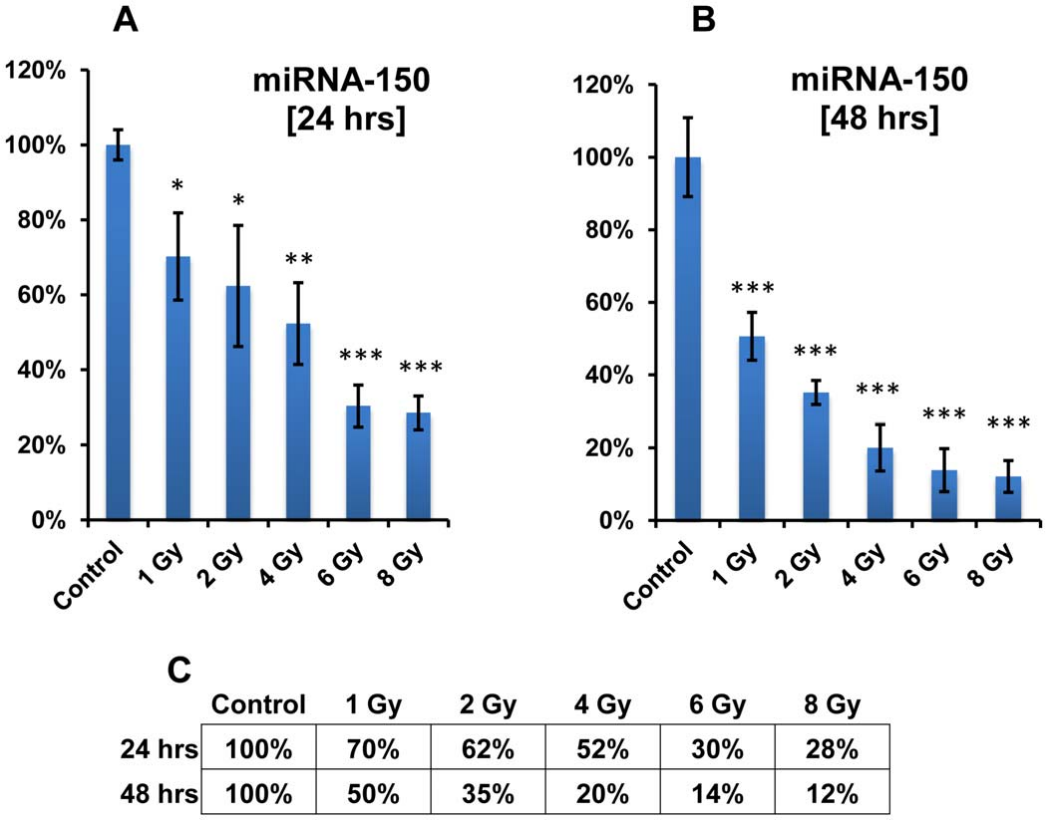
Dose and time dependent depletion of miRNA-150 in animals exposed to 1, 2, 4, 6 and 8 Gy with reference to controls analyzed at 24 hrs (A) and 48 hrs (B). Statistical analysis was performed using an unpaired two-tailed students t-test (*) = *p<0.05*; (**) = *p<0.005;* (***) = *p<0.0005*. C: Kinetics of depletion of miRNA-150 as a function of dose and time relative to respective controls.

miRNA-150 was further investigated for its kinetics of depletion by comparing the dose response at 24 and 48 hrs. A 30% reduction in serum miRNA-150 was observed in animals 24 hrs after 1 Gy total body radiation exposure, which further decreased to 50% by 48 hrs (Figure 6). A time and dose dependent decrease in serum miRNA-150 was evident with an increase in dose, where a gradual decrease in counts was observed with increasing dose. This further confirms the sensitivity and robustness of this serum marker as a candidate for radiation biodosimetry.

### Dose and Time Dependant Changes in miRNAs After Fractionated Radiation Exposure

**Figure 7:**
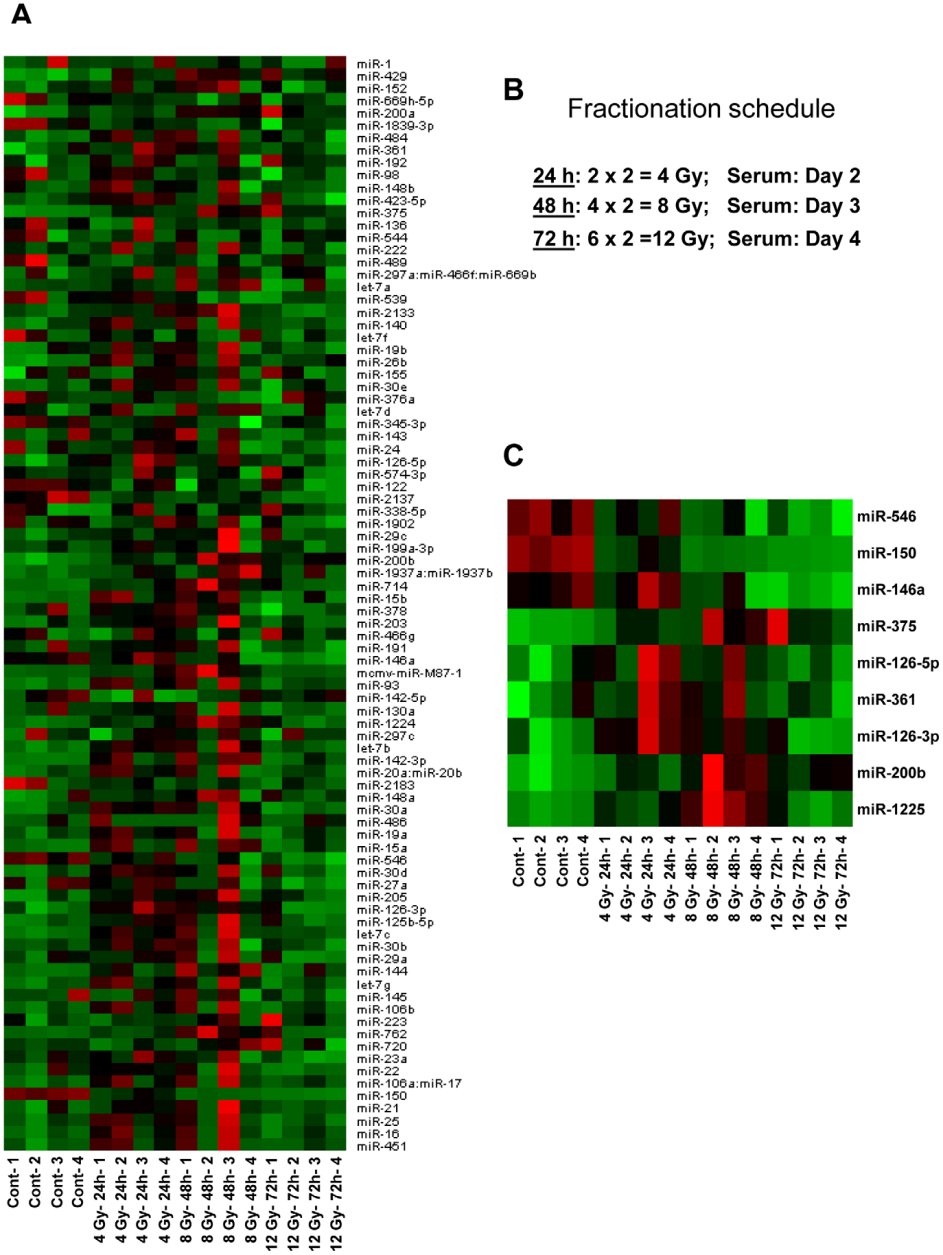
An overview of changes in miRNA levels with fractionated radiation as a function of dose and time. A: The heat map was generated using the normalized data for 88 miRNAs detected in serum from four each of control and irradiated animals collected 24 hrs (2×2 Gy = 4 Gy), 48 hrs (4×2 = 8 Gy) and 72 hrs (6×2 Gy = 12 Gy). B: Scheme of the fractionation schedule. C: A panel of 8 miRNAs selected from ANOVA with a cutoff p-value of 0.05. Red marks high and green marks low expression.

**Figure 8:**
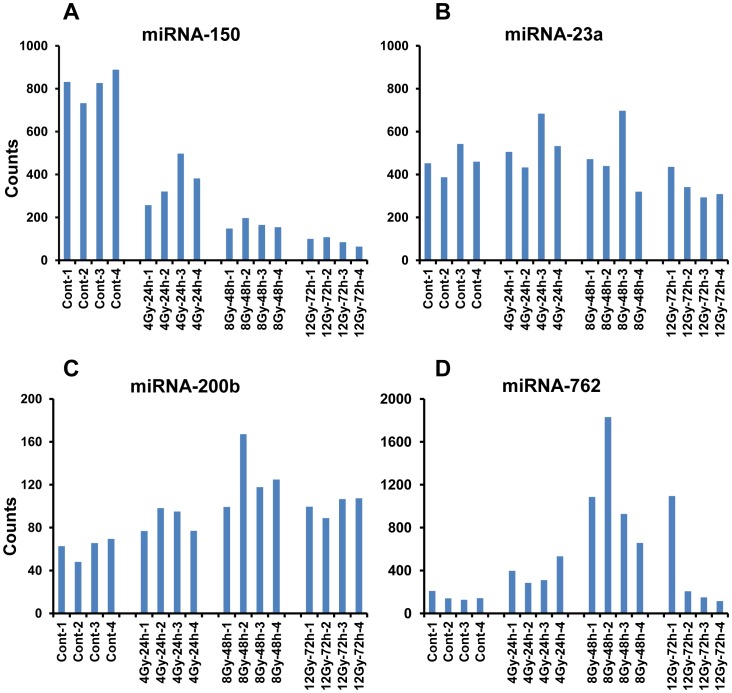
Variations in the counts of selected miRNAs following fractionated radiation. The fluorescent counts obtained after normalization were plotted for individual animals with the dose and time as given. A: The dose dependent depletion of serum miRNA-150 at different time points during and after fractionation (p-values: 4 Gy- 0.0003, 8 Gy- 0.0001, 12 Gy- 0.0001). B: Counts from a non-responsive molecule miRNA-23a (control). C and D: Radiation induced increases in miRNA-200b (p-values 4 Gy- 0.014, 8 Gy- 0.0047, 12 Gy 0.0027) and miRNA-762.

In order to further investigate the biodosimetry potential of the identified miRNAs in the setting of clinical therapeutic radiation, we compared the changes in miRNAs in animals exposed to fractionated doses. Mice were exposed to fractionated radiation following a schedule comparable to that administered to patients receiving total body irradiation as preparative regimen prior to bone marrow transplantation. Twelve week old mice were exposed to a total dose of 4, 8 and 12 Gy in 2 Gy fractions twice a day. Serum collected at 24 hrs (2×2 Gy = 4 Gy), 48 hrs (4×2 Gy = 8 Gy) and 72 hrs (6×2 Gy = 12 Gy) was analyzed for changes in miRNAs using the multiplex nCounter platform (Figure 7). Serum collected from the same animals three weeks prior to radiation exposure was used to compare their basal levels, and the dose and time dependent changes. Moreover, several of those miRNAs that responded to acute single dose was found sensitive to fractionated radiation as well. Consistent with data from single acute dose, about 50% reduction in serum counts for miRNA-150 was observed in mice that received 4 Gy by 24 hrs. A further decrease was observed with higher doses at later time points (Figure 8). Consistent with the response to single acute dose, markers such as miRNA-762 and miRNA-200b exhibited an increase in their serum levels under conditions of fractionated radiation up to 48 hrs. However, a decrease in miRNA-762 was observed at 72 hours. Overall, the data establishes the biodosimetry potential of selected miRNAs under conditions receiving acute single dose as well as fractionated radiation.

## Discussion

The current study has identified several evolutionarily conserved miRNAs responsive to acute radiation in a dose range relevant to accidental radiation exposure or clinical radiation therapy. Identification of serum abundant radio-responsive and non-responsive miRNAs together with spike-in oligos provide a panel of markers and controls for developing radiation biodosimeters. This will aid rapid diagnostic screening to identify individuals who are at risk of developing acute radiation syndromes. Accurate dose evaluation is critical for making medical decisions and timely administration of mitigators to prevent or reduce the acute and late effects. Individual miRNAs such as miRNA-150 alone or in combination with other markers have the potential to estimate the dose to which the individual was exposed. The majority of serum miRNA markers did not respond to radiation, but the hierarchical clustering has identified several markers, potentially originating from blood cells, exhibiting dose- and time- sensitive responses to acute single or fractionated dose. In this study, 24 and 48 hr time points were used, which are realistic time frames to collect blood samples in a scenario involving mass causality from radiation exposure. miRNA-150 depletion kinetics indicate that the response is fast and robust with a near complete depletion in 48–72 hrs with 8 Gy acute dose and 8–12 Gy fractionated dose. The evaluation of the kinetics of depletion of miRNA-150 during three days of fractionation, using a schedule followed in a clinical setting, signifies the translational potential of this marker. In addition to chemo-based approaches, fractionated total body irradiation is used for conditioning in patients undergoing bone marrow transplantation. At the same time, management of hematopoietic injury is a major clinical question in both chemo and radiation based cancer therapies. Recent studies show that miRNA-150 control hematopoiesis [29], [30], [31] and their dose and time dependent changes should provide readouts for the conditioning as well as hematopoietic injury response [31]. Recent genetic studies have shown that overexpression of miRNA-150 results in a slow recovery rate after 5-fluorouracil induced injury in a mouse model for bone marrow transplant [30]. Comparison of serum miRNA-150 from human patients receiving chemo versus radiation based treatments will help evaluate the effects of different conditioning regimens and recovery responses.

We propose miRNA-150 as a sensitive marker for damage to the hematopoietic system, which is the most radiosensitive organ/system. The biodosimetry potential of miRNA-150 is evident from its time and dose dependent depletion, correlating with lymphocyte depletion kinetics [1], [9], [32]. Moreover, miRNA-150 is abundant in serum (ranked among the top 6 miRNAs in serum), and was found to be sensitive even at 1 Gy, the lowest tested dose in the current study. The time and dose response of this marker makes it a potential alternative to complete blood counts and lymphocyte depletion kinetics, the current diagnostic tools for evaluating radiation response. Future experiments would include parallel evaluation of complete blood count and miRNA-150 and comparison of their kinetics. Circulating miRNAs have also been reported from other tissues such as liver and heart under disease states, detected by PCR based approaches [33], [34]. Moreover, the major source of circulating miRNAs is blood cells as evident from their changes as a function of blood cell counts. A recent miRNA profiling from sorted cell types shows lymphoid enriched expression of miRNA-150, whose plasma levels mirrored the lymphocyte counts [35]. Recent RT-PCR based studies have shown that the plasma levels of miRNA-150 can vary under some disease states [36] and inter-individual variations in lymphocyte counts are expected to contribute to changes in basal levels of serum miRNA-150 [12]. Nonetheless, comparing samples collected at multiple time points should provide information on the dose exposed even in individuals with varying basal counts.

Cells are likely in a constant state of releasing miRNAs through secretory vesicles [21], [37], [38], and their level should correlate with the abundance of particular cell types releasing them in the blood stream. The passive release of miRNAs could occur due to tissue injury or systemic response [39]. There are also reports about the ceramide dependent pathways regulating the intercellular transfer of miRNAs [38] and possible release of miRNAs via apoptotic bodies [40]. The variations could also be due to enhanced processing of precursors as well as changes in transcription and their release to the circulatory system. For example, the expression or processing of miRNA-21 is regulated by cytokines such as Tumor Necrosis Factor-α (TNFα) and Transforming Growth Factor 1 (TGFβ1), whose serum levels are also altered as a function of radiation [41], [42]. Radiation is also known to alter TGFβ signaling, which in turn could result in altering the processing of precursors by the DROSHA complex [43]. The stability of miRNAs in serum and plasma could be due to their smaller size and protection in exosomes, microvesicles and Argonaute 2 protein complexes [22], [44], [45]. Studies have shown that miRNAs are stable in serum and plasma at room temperature for hours or even days and after several freeze-thaw cycles [46].

Although the comparison of miRNA in body fluids has tremendous translational potential, especially for early prediction of diseases and evaluation of response to treatments, there were major issues associated with quantification of low abundant extracellular RNA. Since miRNAs are in low abundance in body fluids, PCR based assays do not provide good quantification with acceptable Ct values. Therefore, previous studies relied on templates that were subjected to pre-amplification [15], which incorporate errors and biases under most conditions. There were also technical limitations in the scaling up of starting material, such as limited column capacity and enrichment of contaminants that affect the sensitive enzymatic reaction during downstream analysis. Our study addressed several of these outstanding issues associated with quantitative analysis of extra cellular RNAs as biomarkers. We have used improved normalization methods using multiple validated spike-in oligos in a range comparable to that of the molecules being compared. An amplification-free, hybridization based direct digital counting using specific code sets for individual miRNAs allowed us to determine their relative abundance, with small amounts of RNA derived from small volume of body fluids.
